# Combining Literature Review With a Ground Truth Approach for Diagnosing Huntington's Disease Phenocopy

**DOI:** 10.3389/fneur.2022.817753

**Published:** 2022-02-10

**Authors:** Quang Tuan Rémy Nguyen, Juan Dario Ortigoza Escobar, Jean-Marc Burgunder, Caterina Mariotti, Carsten Saft, Lena Elisabeth Hjermind, Katia Youssov, G. Bernhard Landwehrmeyer, Anne-Catherine Bachoud-Lévi

**Affiliations:** ^1^AP-HP, Hôpital Henri Mondor-Albert Chenevier, Centre National de Référence Maladie de Huntington, Service de Neurologie, Créteil, France; ^2^Univ Paris Est Creteil, INSERM U955, Institut Mondor de Recherche Biomédicale, Laboratoire de Neuropsychologie Interventionnelle, Creteil, France; ^3^Département d'Etudes Cognitives, École normale supérieure, PSL University, Paris, France; ^4^Movement Disorders Unit, Institut de Recerca Sant Joan de Déu, CIBERER-ISCIII, Barcelona, Spain; ^5^European Reference Network for Rare Neurological Diseases (ERN-RND), Tübingen, Germany; ^6^Siloah and Department of Neurology, Department of Clinical Research, Swiss Huntington's Disease Centre, University of Bern, Bern, Switzerland; ^7^Unit of Genetics of Neurodegenerative and Metabolic Diseases, Carlo Besta Neurological Institute IRCCS Foundation, Milan, Italy; ^8^Department of Neurology, Huntington Center North Rhine-Westphalia, Ruhr-University, St. Josef-Hospital, Bochum, Germany; ^9^Department of Neurology, Rigshospitalet, Danish Dementia Research Centre, Clinic of Neurogenetics, Copenhagen University Hospital, Copenhagen, Denmark; ^10^Department of Neurology, University of Ulm, Ulm, Germany

**Keywords:** Huntington's disease, chorea, phenocopy, diagnosis, differential diagnosis, guidelines, daily clinical practice

## Abstract

One percent of patients with a Huntington's disease (HD) phenotype do not have the Huntington (HTT) gene mutation. These are known as HD phenocopies. Their diagnosis is still a challenge. Our objective is to provide a diagnostic approach to HD phenocopies based on medical expertise and a review of the literature. We employed two complementary approaches sequentially: a review of the literature and two surveys analyzing the daily clinical practice of physicians who are experts in movement disorders. The review of the literature was conducted from 1993 to 2020, by extracting articles about chorea or HD-like disorders from the database Pubmed, yielding 51 articles, and analyzing 20 articles in depth to establish the surveys. Twenty-eight physicians responded to the first survey exploring the red flags suggestive of specific disease entities. Thirty-three physicians completed the second survey which asked for the classification of paraclinical tests according to their diagnostic significance. The analysis of the results of the second survey used four different clustering algorithms and the density-based clustering algorithm DBSCAN to classify the paraclinical tests into 1st, 2nd, and 3rd-line recommendations. In addition, we included suggestions from members of the European Reference Network-Rare Neurological Diseases (ERN-RND Chorea & Huntington disease group). Finally, we propose guidance that integrate the detection of clinical red flags with a classification of paraclinical testing options to improve the diagnosis of HD phenocopies.

## Introduction

Huntington's disease (HD) is the most frequent inherited chorea in adults (1–3), with an estimated prevalence of about 10 individuals per 100,000. Its clinical phenotype is defined by a triad of symptoms and signs (3):

(1) A motor syndrome encompassing a wide range of symptoms, including pyramidal and movement disorders. The essential feature, chorea, is defined by excessive, spontaneous, irregularly timed, non-repetitive, randomly distributed, and abrupt movements which may affect the face, the trunk, and extremities.
(2) The cognitive symptoms include impaired executive functions or apathy in the early states.
(3) Psychiatric signs include a wide range of symptoms such as irritability, anxiety, or depression. Cognitive and psychiatric symptoms may appear as prodromal symptoms years before diagnostic motor signs.

About 1% of patients with this clinical phenotype do not have CAG expansion in *HTT* (1); they are described as HD phenocopies. An HD phenocopy is defined as (2):

1) a movement disorder consistent with HD when assessed by an experienced neurologist.
2) a negative test for the pathogenic CAG repeat expansion in *HTT*.
3) a family history suggestive of autosomal dominant inheritance, cognitive impairment, behavioral, or psychiatric symptoms.

Determining the root cause of HD phenocopies is difficult due to a large number of disease entities giving rise to a HD-like clinical presentation, with an ever increasing number of conditions, most markedly in the last decade (4, 5) (Table 1). However, some of these disease entities may exhibit unusual signs highly suggestive of a particular disease, which may serve as “red flag” signs to guide the work-up including the choice of paraclinical tests. Although the concept of red flags is intuitive for physicians (12), only one article explicitly applies it to the etiologic diagnosis of chorea syndromes (13). In addition, the specific paraclinical tests deemed necessary are sometimes unaffordable or inaccessible. Therefore, we propose a study combining a literature review aimed at identifying the red flags useful for the diagnosis of chorea and a survey of chorea experts, to validate the feasibility and usefulness of these choices based on a ground-truth approach. When combined with a literature review, this approach would help define a rational diagnostic strategy for HD phenocopies in daily clinical practice (DCP).

**Table 1:** Diagnoses found in adults with non-Huntington chorea (2, 4–11).

**Autosomal dominant**	**Main genes**
Spinocerebellar Ataxia (SCA, Dentatorubral-pallidoluysian atrophy)	*TBP, ATN1*
Huntington disease like 2	*JPH-3*
C9ORF72 mutation	*C9ORF7*
PRNP mutation: Inherited prion disease/HDL1	*PRNP*
Benign hereditary chorea	*NKX2.1 /TITF1*
NBIA Neuroferritinopathy	*FTL1*
Fahr's disease: basal ganglia calcification	*SLC20A2, PDGFRB, PDGFB, JAM2, MYORG, XPR1*
**Autosomal recessive**	
Friedreich Ataxia	*FXN*
Ataxia telangiectasia	*ATM*
Ataxia with oculomotor apraxia (AOA1, AOA2)	*APTX, SETX*
Chorea-acanthocytosis	*VPS13A*
Wilson's disease	*ATP7B*
NBIA (Aceruloplasminemia, PKAN, PLAN)	*Ceruloplasmin CP, PANK2, PLA2G6*
Niemann Pick type C	*NPC1*
**X linked and mitochondrial**	
McLeod neuroacanthocytosis syndrome	*XK*
Lubag syndrome (DYT3)	*TAF1*
**Sporadic**	
Drug	
Metabolic disorders (glycemia, B12, thyroid, parathyroid, calcium…)	
Autoimmune (lupus, Sjögren, antiphospholipid, Celiac disease…)	
Cerebrovascular cause	
Paraneoplastic (anti-CRMP5/CV2, anti-NMDA receptors…)	
Syndenham's chorea	
Infectious disease: HIV, syphilis infection…	

*NBIA, Neurodegeneration with Brain Iron Accumulation; AOA, Ataxia with Oculomotor Apraxia; PKAN, Pantothenate kinase-associated neurodegeneration; PLAN, PLA2G6-Associated Neurodegeneration; HIV, human immunodeficiency virus; anti-CRMP5/CV2, collapsin response-mediator protein-5 antibodies; anti-NMDA, N-methyl-D-aspartate receptor antibodies*.

The aim of our study was to provide a combined approach based on physician expertise associated with a literature review that would enable diagnosing the patient with HD phenocopy.

## Methods

**Figure 1:**
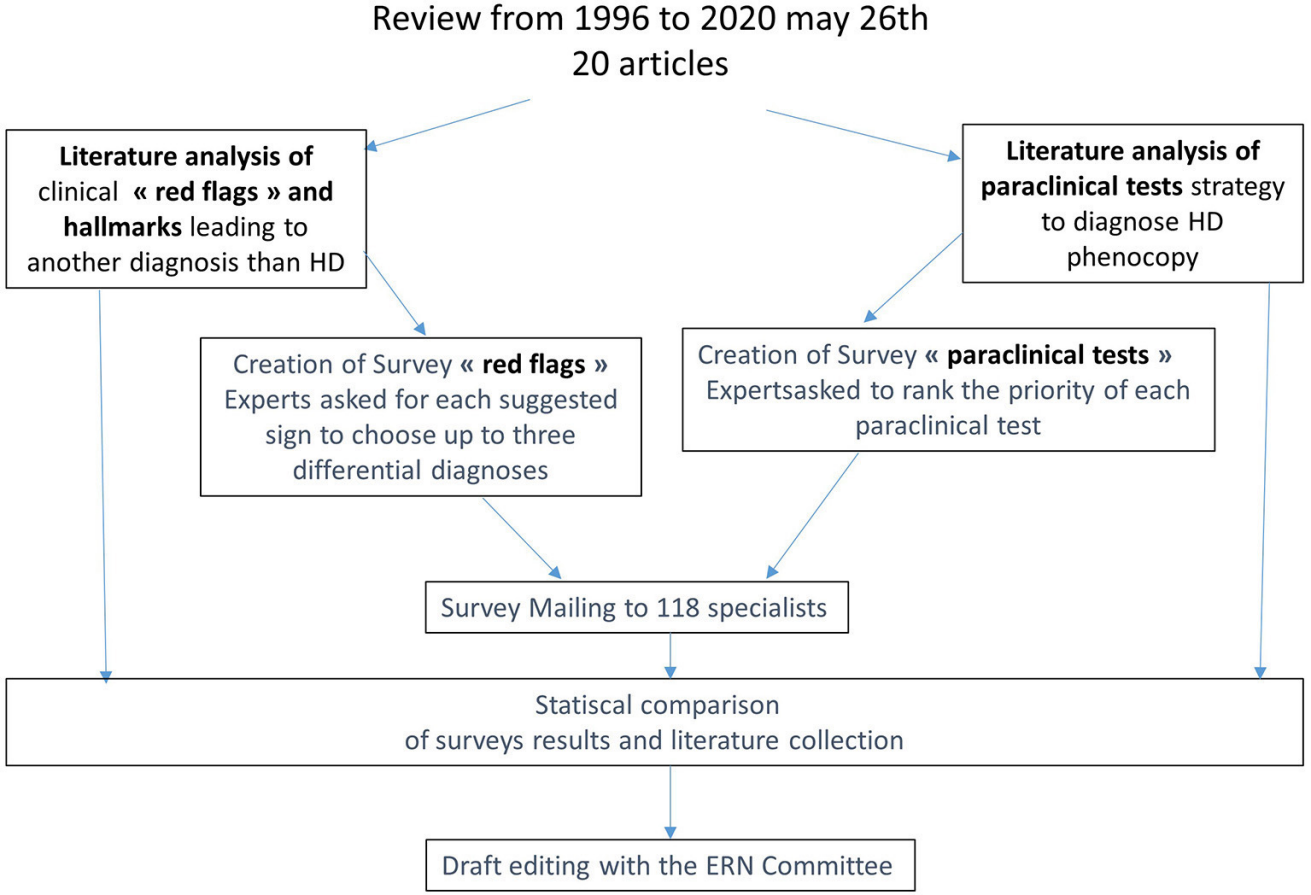
Two complementary approaches, literature review, and surveys, to identify useful red flags and paraclinical tests to guide diagnosis.

We used a sequential three-step approach: (a) a review of the literature; (b) a survey to assess DCP; and (c) the inclusion of the European Reference Network-Rare Neurological Diseases (ERN-RND) group suggestions (Figure 1).

### Literature Review

To design the surveys and identify potentially red flags in DCP, we performed a PubMed review from 1993 onwards, when the HD mutation was first identified, to May 26, 2020. The query was formulated as follows: ((“Diagnosis” [MeSH]) AND (“chorea” [MeSH Major Topic])) AND (“Huntington Disease” [MeSH])). We selected articles with the keywords “Chorea” or “Huntington^*^” in the title and restricted our search to papers dealing with diagnostic issues. For example, titles dealing with biomarkers, animal models, or medical care were excluded from the search. As a result, we selected 51 abstracts.

They were forwarded to two neurologists (KY and ACBL) to identify whether they actually dealt with the diagnosis of phenocopies. A third neurologist (QTRN) arbitrated in cases of disagreement between the first two. As a result of this selection, 20 articles (2, 4, 6–11, 13–24) were extracted and analyzed.

### Analyzing the Literature Review

Red flags were self-evident when they appeared in a dedicated table. Otherwise, we extracted them from:

- A figure or a specific table presenting a disease-related sign.
- A table summarizing a particular diagnosis with some descriptive signs.
- The main text, particularly in the paragraphs discussing different diseases.

Similarly, information on ordering a paraclinical test could be found when it appeared in an ordered list or in the description of the initial workup, or elsewhere in the main text. We considered a paraclinical test to be 1st-or 3rd-line when it was recommended to be used in all cases or in specific situations only, respectively. In a few cases, certain paraclinical tests were recommended when the first-line tests were negative, which was labeled as a 2nd-line test.

### Creation of Surveys

To collect expert opinions for chorea diagnosis, we created two surveys (https://www.surveymonkey.com/).

A first survey identified clues that would help diagnose in DCP. It comprised 73 questions based on the literature review. For each of the different clinical signs and paraclinical tests identified in the literature, we asked physicians to select from a drop-down menu the first disease entities coming to their mind based on their own experience. They could select up to three diagnoses or answer “no suggestion” if none of them applied to their practice.

A second survey was aimed at prioritizing 43 paraclinical tests identified in the literature review for assisting in the diagnosis of HD phenocopies. We asked physicians to categorize each test into one of three groups: the 1st-line tests (to be ordered under all circumstances), the 2nd-line tests (if 1st-line tests are inconclusive), and the 3rd-line tests (only done in specific cases).

In both surveys, physicians could fill out open-ended comment boxes. We also added to our list of terms for surveys some signs and paraclinical tests in DCP too obvious to mention in the literature review, such as “arthritis” or “thoracic-abdominopelvic CT scan.”

The surveys were submitted online from May 2018 to May 2019 to 118 physicians (neurologist and geneticist) (25) and members of the ERN-RND. In October 2019, two reminders were sent to get a full response. Analyses started in December 2019.

### Analysis of the Surveys

We calculated online (https://www.surveymonkey.com/mp/margin-of-error-calculator/) and applied the margin of error with a confidence interval of 95% to the results of the surveys. For each red flag, we added up the total number of votes for the three top diagnoses indicated by the physicians.

For the paraclinical tests survey, the sum of the 1st, 2nd, or 3rd-line votes gave three dimensions that were analyzed in four different clustering algorithms to ensure the stability of the result (using the scikit-learn module with Python). K-means and Gaussian mixtures were run 100 times to minimize the bias due to chance. We also used the deterministic DBSCAN algorithm and agglomerative hierarchical clustering, which were run four times with different parameters. We then summed the results of these four algorithms to classify the paraclinical tests into three prescription lines.

Finally, we added additional suggestions from the ERN-RND Chorea & Huntington disease group.

## Results

### Description of the Literature Review Process and the Respondents

The literature review ended-up with 20 articles out of 49 abstracts (see the Flow diagram in Supplementary Material). All were review articles but six that were series of case description [cases (17, 19), familial (14, 15), or cohort (18, 22) descriptions]. More specifically, one single article employed the red-flag concept to guide the investigations (13). Additional red flags were extracted from figures or tables from eight articles (2, 6, 9, 11, 13, 19, 20, 23). The strategy for ordering paraclinical test were synthetized from ordered lists in four articles (2, 8, 9, 20) and in workups proposed in five additional articles (2, 6, 16, 19, 23). Additional information was extracted from the main text of all selected articles.

Twenty out 28 physicians registered for the red flag survey and 25 out 33 for the paraclinical survey, with 17 responding to both completed the survey. All participants were European except one (United States) for the red flag survey and three (one each from Australia, China, and the United States) for the other survey.

### Red-Flags

Forty-three percent of the 73 questions reached a participation rate of >75%, with a minimum participation of 60%. Sixty-seven answers obtained a majority vote (>50%), with 17 exceeding 75% of votes. Twenty red flags were not attributed to any diagnosis by majority vote. With a sample size of 20 out of the 118 physicians, the voting results had a margin of error of 20%.

Table 2 summarizes the main results of the survey with votes that exceeded 30%. Some disease entities were voted for significantly by more than 70% of the physicians, and thus achieved 50% of the votes despite a 20% margin of error: HDL2 in African ancestry (75%), DRPLA in Japanese ancestry (85%), cerebrovascular disease in acute cases onset (74%), or with unilateral signs (93%), Mc Leod in myopathy (73%), or acanthocytosis (71%), Chorea-acanthocytosis (ChAc) in Acanthocytosis (100%) or increased creatine kinase (71%), DRPLA in myoclonus (80%), SCA17 in severe cerebellar ataxia, Wilson's disease in Kayser-Fleischer sign and ascites (100%), liver failure or increased liver enzymes (71%), Ataxia-telangiectasia in telangiectasia (92%), or increased alpha-protein (86%), AOA in oculomotor apraxia (86%), PKAN in the eye of the tiger sign (77%). Diagnostics which votes were below the 30% threshold in the survey were still mentioned in the literature column when emphasized in the review.

**Table 2:** Red flags identified in the survey and literature, as well as suggested diagnoses.

**Cue**	**Red flag**	**Survey**	**Literature**	**Cue**	**Redflag**	**Survey**	**Literature**
Ethnicity	African ancestry	HDL2 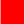		Ophthalmological signs	Kayser-Fleischer sign	Wilson's disease 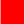	
	Japanese ancestry	DRPLA 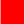 , SCA17			Retinopathy		PKAN, Aceruloplasminemia
	Caucasian ancestry	**C9ORF72**	**SCA8 (Finland)**		Telangiectasia	A. telangiectasia 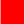	
			**AOA2 (Fr Canad)**		Oculomotor apraxia	**AOA1**	A. telangiectasia
Age of Onset	Childhood	Benign Ch., Sydenham's Ch.				**AOA2**	
					Other oculomotor impairment	**NPC**	
		Infectious disease		Cardiac signs	Cardiomyopathy	Friedreich Ataxia	
		A. telangiectasia				Mc Leod	
		Metabolic disorders				Ch. Acantocytosis	
Evolution mode	Acute	Cerebrovascular cause 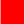			Valvulopathy	**Sydenham's Ch**.	
		**Syndenham's Ch**.				**Autoimmune**	
		Infectious disease			Carditis	Sydenham's Ch., **Autoimmune**	
	Subacute	**Paraneoplastic**		Digestive signs	Ascites, liver failure	**Wilson's disease** 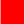	
		**Autoimmune**			Organomegaly	**NPC**	
		**Sydenham's Ch**.				**Metabolic disorders**	Ch. Acanthocytosis
	paroxysmal	**Drug, Metabolic**	Parox. dyskinesia	Pulmonary signs	Pulmonary disease		Benign Ch.
Prominent sign localization	Orofacial and tongue	Ch. Acantocytosis 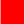	Mc Leod	Osteo-articular signs	Arthritis	**Syndenham's Ch**.	
		Drug	Paraneoplastic			**Infectious disease**	
		PKAN	Hep. Degen.			**Autoimmune**	
	Dysphagia	PKAN			Scoliosis	Friedreich Ataxia	
	Axial		Ch. Acantocytosis	Other	Diabetes	Friedreich Ataxia	Aceruloplasminemia
	One-sided signs	Cerebrovascular cause 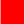	**Struct. Lesion**		Thyroid disease	Benign Ch	
Peripheral signs	Neuropathy	Friedreich Ataxia	Mc Leod			**Autoimmune**	
		Ch. Acantocytosis			Immune deficiency	A. telangiectasia	
	Myopathy	Ch. Acantocytosis				**Autoimmune**	
		Mc Leod 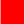			Malignancy	A. telangiectasia	
		**Metabolic disorders**				**Paraneoplastic**	
Epilepsy	Seizures	DRPLA	Mc Leod	Blood cells	Anemia	**Autoimmune**	Mc Leod
		Ch. Acantocytosis	**HDL1**		Acanthocytosis	Ch. Acantocytosis	HDL2
		**Cerebrovascular**	**SCA17**			Mc Leod	PKAN
	Myoclonus and myoclonic seizures	DRPLA	SCA17				**Aceruloplasminemia**
			**C9ORF72**		Hyperleucocytosis	Infectious disease	
			**HDL1**	Biochemical analysis	Increased liver enzymes	**Wilson's disease** 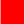	Ch. Acanthocytosis
			**HDL2**			**Metabolic disorders**	Mc Leod
			**Mitochond. dis**.			**Niemann Pick C**	
			Ch. Acantocytosis		Incr. creatine kinase	Ch. Acantocytosis 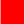	
Other prominent neurological signs	Early mental retardation	NPC**, Metabolic**	DRPLA			Mc Leod	
	Behavioral/cognitive impairment	C9ORF72	FLTD		Increased alpha-foetoprotein	A. telangiectasia 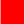	
	Psychiatric prodromes	C9ORF72				AOA2	
	Severe cerebellar ataxia	SCA17 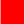	**HDL1**	Brain imaging	T2*/SWI signal in basal ganglia	Neuroferritinopathy	
		DRPLA	AOA			**PKAN**	
		Friedreich Ataxia	Ch. Acantocytosis		T2 hypersignal in basal ganglia	**Wilson's disease**	
		A. telangiectasia	Mc Leod				
			Mitochond. dis.		Eye of the tiger sign	PKAN 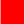	
	Severe oculomotor impairment	AOA1				**Neuroferritinopathy**	
		NPC			Caudate head, basal ganglia atrophy	**HDL2**	
		A. telangiectasia			Cerebellar and brainstem atrophy	SCA17	
		AOA2				DRPLA	
	Severe dystonia	Lubag syndrome	**SCA**			**AOA2**	
		PKAN	**DRPLA**		FLAIR/diffusion in cortex/basal ganglia	**PRNP**	
		Wilson's disease	**Ch. Acantocytosis**				
		Neuroferritinopathy					
	Severe Parkinson	PKAN	NBIA				

*The survey column summarizes the main diagnoses voted on with >30% of the physicians. The literature column showed the diagnoses found in literature but not already mentioned in this table. Bold indicates either diagnoses that were highlighted by the physicians but were not in the literature corpus, or diagnoses that have been highlighted in literature but have not been voted on by physicians. Diagnoses marked with an 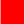 were voted on by >70% of the physicians. HDL, Huntington Disease-Like; DRPLA, Dentatorubral-Pallidoluysian Atrophy; SCA, Spinocerebellar Ataxia; Fr Canad, French Canadian; Ch., chorea; A. telangiectasia, Ataxia Telangiectasia; Parox. Dyskinesia, Paroxysmal Dyskinesia; PKAN, Pantothenate kinase-associated neurodegeneration; Hep. Degen., hepatocerebral degeneration; Struct. Lesion, Other structural lesions; Mitochond. dis., Mitochondrial diseases; NPC, Niemann Pick Disease Type C; FLTD, Frontotemporal lobar degeneration; AOA, Ataxia with Oculomotor Apraxia; NBIA, Neurodegeneration with brain iron accumulation; Severe Parkinson, Severe Parkinsonism; PRNP, prion protein gene mutation*.

Five red flags (axial predominance, upper limb, lower limb, absence, partial seizures, myoclonic seizures, and restrictive lung disease) reached more than 70% “no suggestion” response.

### Paraclinical Tests

**Figure 2:**
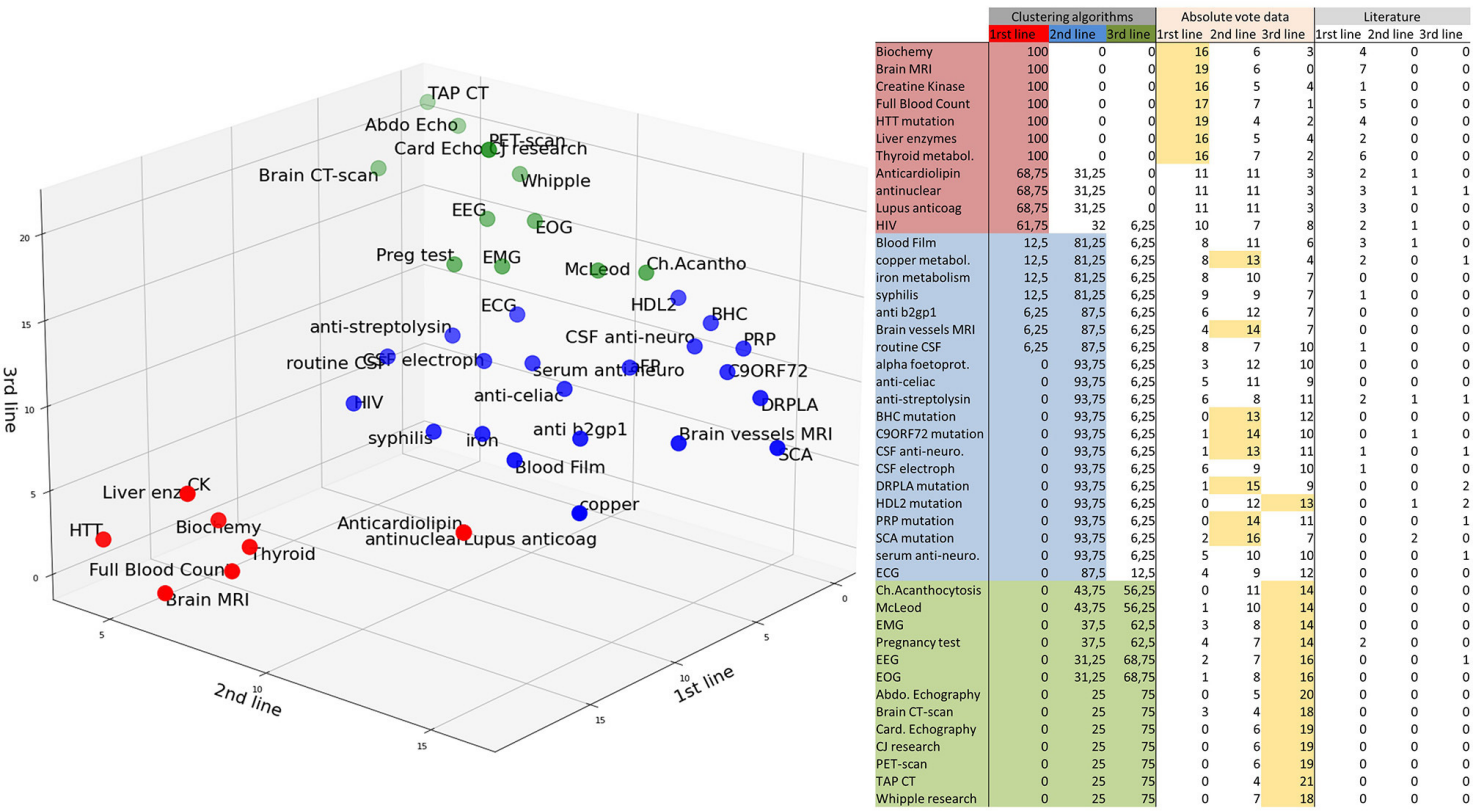
Classification of the paraclinical tests. 1st-line: red, 2nd-line: blue, 3rd-line: green. The results of the clustering algorithms are summed in the left columns (results in % of runs of the algorithms). Absolute numbers of votes are reported in the middle columns as “Absolute vote data.” The majority vote (>50% of 25 votes) is highlighted in yellow. Literature results are summarized in the right columns. McLeod: exclusion of the McLeod Kell phenotype by a blood bank (weak Kell antigens and, if available, no reaction with anti-Kx). Ch.acanthocytosis: Western blot for chorein protein.

Overall, the four clustering algorithms performed similarly. The two non-deterministic algorithms, Gaussian mixture and K-means, resulted in similar clusters. Four tasks were equally classified as 2nd- or 3rd-line tests by the agglomerative hierarchical clustering algorithm: pregnancy tests, electromyograms, and electrocardiograms. The DBSCAN has been optimized to not exclude any data by aggregating the paraclinical tests into two groups. Results are summarized in Figure 2, while adding the absolute number of votes. Brain MRI, complete blood count, biochemistry tests, liver enzymes, creatine kinase, thyroid test, HTT mutation were ranked first. Antinuclear tests, anticardiolipin antibodies, lupus anticoagulant tests, and HIV tests, not clearly ranked high when considering the absolute number of votes, were classified as 1st-line tests. About half of the 2nd-line tests were clearly ranked thanks to the clustering algorithms, and would not if we considered only the absolute number of votes.

## Discussion

**Figure 3:**
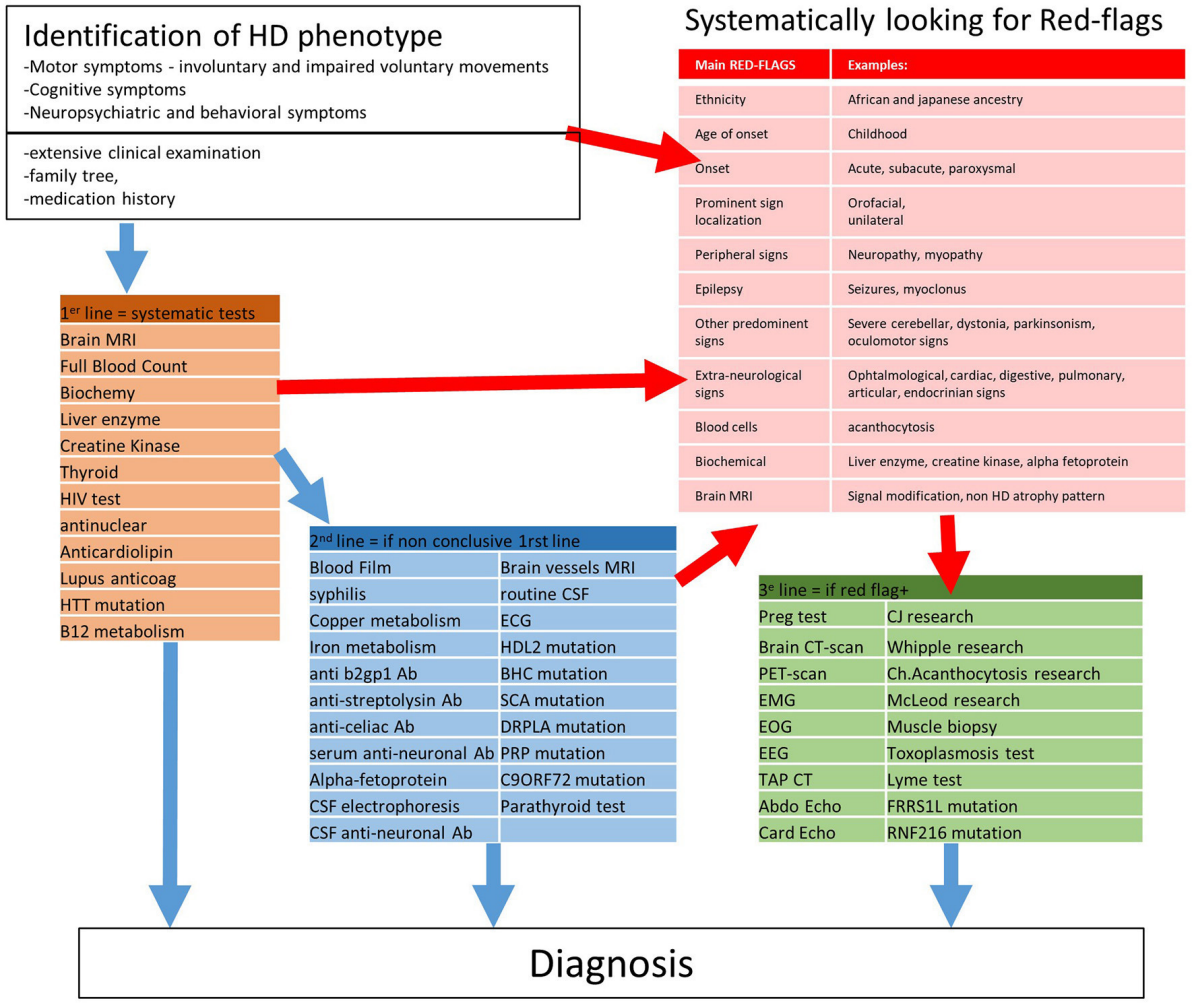
Proposition of a strategy to diagnose HD phenocopy. Ab, Antibodies.

Our study combined a literature review with DCP to provide a ground truth exercise for the diagnostic process (Figure 3). We first identified potential red flags and paraclinical tests in the literature, then constructed two surveys. The first one to validate useful potential red flags in DCP and was answered by 28 experts; the other one on the classification of paraclinical tests in 1st, 2nd, and 3rd-lines was answered by 33 experts and analyzed by clustering algorithms. The results were submitted to the ERN-RND group for final suggestions. We summarized the results of this three-step approach in Figure 3: A diagnostic workup involving around 30 HD phenocopies, with a focus on the 25 most critical red flags, and 50 paraclinical tests sorted into three lines of prescription.

Our quantified strategy provides a pragmatic and relatively comprehensive approach to the differential diagnosis of chorea syndromes. It allows prioritization of the growing list of HD phenocopies that are difficult to integrate into DCP. It allowed us to expand the list of phenocopies compiled through several literature reviews (2, 11, 13–16) while still prioritizing (identifying 12–23 red flags and 5–26 unsorted paraclinical tests for 10–32 disease entities discussed). Our approach, combining field surveys with a literature review, reflects current practice and integrates the availability and real-life feasibility of the examinations and thus identify the true root cause of chorea syndromes. We have thus identified clinical and paraclinical alarm signals (Table 2) pointing to a handful of diagnoses. Being more targeted than in a classic review, the choice of proposed examinations should help toward reducing the cost of the diagnostic workup to society and the discomfort for patients. Physicians tend to order as many paraclinical tests as possible when faced with an unsolved diagnostic problem. Often, the cost-benefit ratio is unknown and not available in the WHO scope for neurological diseases. Common sense would suggest, however, that the best diagnostic process is one in which the physician directly targets the true root cause of a chorea syndrome and correctly identifies the most appropriate choice of paraclinical tests to confirm the presence of a distinct disease entity. This approach may be more limited in scope when advances in genetics offer panels of genes targeted to chorea in a single assay. However, not all chorea is genetic, and the clinical approach guided by red flags (Table 2) and lists of “cost-effective” tests continues to be a reasonable approach for clinicians in the diagnostic process.

Our results emphasizes the importance of a thoughtful clinical examination (including visual exploration, neuropathy assessment, and a search of other associated neurological and non-neurological features) to comprehensively ascertain the HD phenotype and potential red flags as a first step toward diagnosis. One should always evaluate the inheritance (Table 1) with a family tree and carefully investigate the psychiatric family history. The HD expansion mutation should be searched for as first-line test in any HD phenocopy. However, genetic counseling is mandatory not only before ordering molecular test for HD but also for any genetic diagnosis, to prepare patients for a possible unfavorable result. At this point, drug-induced chorea should also be ruled out. Then, based on red flags, we can plan the paraclinical investigations along the three lines (Figure 2). In all patients, a neurologist should order the 1st-line test, the results of which are frequently obtained before the results of the HD mutation. Then, one should consider 2nd-line tests in case the 1st-line tests are inconclusive. Our 1st and 2nd-line tests were consensus in the surveys and cover most of the curable and frequent HD phenocopies. Red flags should guide more specific 3rd-line testings.

### Clinical Features

#### Ethnicity

As a result of the predominance of HD in the Caucasian population, ethnicity was useful when no expansion mutation in *HTT* was identified. A recent study of black South African patients with HD-like phenotype showed that 31% had CAG expansion in *HTT*. In the group of patients negative for the *HTT*-expansion mutation, 13% had HDL2, 1% SCA2, and no C9ORF72 mutation (26). HDL2 is a perfect HD phenocopy both clinically and radiologically and should therefore be considered in HD phenocopies with African ancestry.

The prevalence of HD is about 10 times lower in Asian populations than in the European populations (3). In a Japanese study (27), HD was found in 46% of patients with chorea, and 16% of patients negative for the *HTT*-expansion mutation had a Dentatorubral-Pallidoluysian Atrophy (DRPLA) mutation. Lubag's syndrome is another example: patients had a Filipino ancestry and an X-linked dystonia-parkinsonism syndrome, in which some female carriers may also exhibit chorea (28).

#### Age at Onset

It is also important to consider the age at onset since some diseases may begin in childhood (29) but are not diagnosed until the patient is an adult, either in the case of an atypical adult-onset disorder or in childhood-onset diseases with persistent chorea. Benign Hereditary Chorea (BHC) (30), characterized as early-onset chorea in childhood (usually 2–3 years old), remains stable or usually improves in adolescence and adulthood, hence its name. Its motor phenotype is often preceded by hypotonia or motor developmental delay within the first year of life (30) associating other movement disorders such as ataxia, dystonia, or tics. The remaining key features of HD phenotype (cognitive and psychiatric) were previously considered as mild and rare in contrast to other conditions (31). However, cognitive assessment often discloses attention deficit hyperactivity disorder and reduced IQ (30), along with various psychiatric symptoms (such as obsessive-compulsive disorder or psychosis). The NKX2.1 gene mutation, found in BHC, is also related to a broader non-neurological clinical spectrum reported as the “brain-lung-thyroid” syndrome: typically, neonatal, or early respiratory distress, recurrent respiratory infections, and asthma along with congenital hypothyroidism. Several other developmental disorders and a propensity for malignancy have also been reported (32).

There are about 100 genes associated with childhood-onset chorea. Some forms are usually lethal before adulthood. However, atypical cases of patients with adult-onset movement disorder have been described. For a better description of these genes, the ERN-RND childhood-onset chorea algorithm available at http://www.ern-rnd.eu/wp-content/uploads/2019/10/Diagnostic-flowchart-for-Childhood-onset-Chorea.pdf may be consulted.

#### Progression and Mode of Onset

An acute onset, especially if chorea and other neurological signs are unilateral, should prompt us to direct investigations toward an acute vascular etiology. Hemiballism is classically described but can speedily diminish in days.

A rapidly progressive phenotype affecting the entire central nervous system (including cognitive decline, visual, cerebellar, pyramidal and extrapyramidal disturbance with myoclonus, sometimes insomnia) should raise the possibility of prion disease. Its highly variable phenotype, which includes movement disorders and chorea (11%) (33), implies other specific signs like MRI abnormalities (deep nuclei and cortical hyperintensities), elevated 14-3-3, and tau protein in CSF and an abnormal EEG (typical periodic sharp wave complexes). The literature usually classifies prion disease as sporadic Creutzfeldt-Jakob disease (sCJD), acquired, or dominant inherited prion disease [historically called HDL-1 (14)]. The latter could be misdiagnosed as HD, since the progression may be slower, and the patients can also present personality changes (34).

Paroxysmal chorea should be considered a red flag for rare paroxysmal movement disorders (35), most notably paroxysmal dyskinesia, which is characterized by attacks of choreatic or dystonic movements. Secondary causes (such as metabolic, auto-immune, or paraneoplastic) should be considered rather than genetic causes in adults. Inherited paroxysmal dyskinesia are grouped into kinesigenic, non-kinesigenic, or exercise-induced dyskinesia. Diagnosis can be difficult as there may be no interictal neurological and cognitive signs depending on the disease entity.

#### Cerebellar Signs, Dystonia, and Parkinsonism

In our survey, cerebellar ataxia, a predominance of dystonia, and parkinsonism did not much contribute to diagnosing defined chorea syndromes. Their low specificity is reflected by a scattered distribution of the votes, with some diagnoses falling below the 30% threshold. Although these signs are found in most of the diseases involving chorea, including HD, and thus were less informative red-flags, these still need to be assessed.

These symptoms could be observed accompanying chorea in inherited ataxia (36). Among autosomal recessive ataxias, Friedreich's ataxia (FA) starts relatively late (after 25 years); the presence of chorea is rare. FA should be suspected when an HD phenotype is associated with areflexia, progressive ataxia, hypertrophic cardiomyopathy, scoliosis, and diabetes. Oculocutaneous telangiectasia usually develops at a young age in ataxia-telangiectasia (AT). It may be associated with a high alpha-fetoprotein level and other suggestive symptoms such as ataxia, neuropathy, or oculomotor apraxia. Oculomotor apraxia, defined by a difficulty in moving the eyes when ordered without any motor impairment, in other autosomal recessive ataxia: ataxia with oculomotor apraxia (AOA) type 1 and 2. Chorea is even more frequent than ataxia in AOA1 compared to AOA2 or FA (37). Patients with AOA also exhibit sensorimotor neuropathy.

Spinocerebellar Ataxia (SCA) is a large family of autosomal dominant ataxias with a variable association of signs, including gait ataxia, neuropathy, epilepsy, pyramidal, cognitive, and psychiatric symptoms, but also signs of basal ganglia dysfunction such as chorea, dystonia, or parkinsonism (38). SCA 17 has a particular phenotype with prominent dementia and chorea, thus known as “HDL 4.” Other SCA should not be forgotten, however chorea can be observed during the course of several SCAs though as a rule less frequently than in SCA 17 (39). DRPLA is variably associated with the phenotypes of SCA, HD, and myoclonic epilepsy (40).

#### Cognitive and Psychiatric Signs

Although critical to the evaluation of patients with an HD-like phenotype, behavioral, cognitive, and psychiatric symptoms did not appear to be specific enough to identify different diagnoses. Both our investigations and the literature review suggested numerous diagnoses for these clinical signs, without being able to point to any specific one. It could be due to unspecific wording of the cognitive and behavioral defects. Some severe symptoms are less common and should lead to consider a behavorial variant of frontotemporal degeneration (FTD) (41): predominant behavioral disturbance, significant personality change, violation of social rules, loss of empathy, carbohydrate hyperphagia, or aphasia in its language variant. There are reports on patients with an FTD phenotype associated with a relatively pure choreatic syndrome. In such cases, neuroimaging may help identify this behavioral phenotype by demonstrating frontotemporal lobar atrophy. TAR DNA-binding protein 43 (TDP-43) pathology (41–43) or fused in sarcoma (Fus) (44) were more frequent than tauopathies, which may be seen in HD as well (45). We did not discuss in our study the corresponding mutations [such as TAR DNA binding protein (TARDBP), progranulin, or microtubule associated protein tau (MAPT)] that should be ordered, especially with a suggestive family history.

There is also emerging evidence of the role of a C9ORF72 mutation. This mutation is usually associated with FTD or atrophic lateral sclerosis (ALS) in the Caucasian population (46), and was mentioned as the first HD phenocopy, above SCA17, in an English cohort (19). It remains difficult to determine the pathogenic number of repeats, notably as large expansions are not uncommon in the UK population (47). Altogether, we suggest that C9ORF72 should be considered in the 2nd-line tests, especially if there is a personal or familial history of ALS or FTD.

### Paraclinical Features

#### Blood Tests

Most metabolic disorders associated with chorea can be easily ruled out by simple blood tests. This comprises electrolyte disorders including dysglycemia, hypocalcemia, and dysnatremia, as well as systemic metabolic disorders such as renal or hepatic failure, and hyperthyroidism. Vitamin B12 depletion was described in chorea syndromes, along with a recovery following B12 replenishment (48). An elevated hemoglobin and hematocrit suggesting polycythemia vera should not be missed (49).

When acanthocytosis is found in a peripheral blood smear, the focus should be on Neuroacanthocytosis (NA) diseases, which include both Chorea-Acanthocytosis (ChAc) and McLeod Syndrome (MLS). Clinically, both major NAs are present with neuropsychiatric and cognitive symptoms, seizures, neuromuscular symptoms (such as muscle atrophy or areflexia), or increased creatine kinase. In ChAc, movement disorders are often axial disturbances in gait and posture, with orofacial signs including dysarthria, vocalization, tongue protrusion, and feeding dystonia (13). In MLS, cardiac manifestations must be carefully considered and monitored. Acanthocytosis can also be seen in other phenocopies such as HDL2, PKAN, or aceruloplasminemia.

As a potentially treatable condition, one should always consider autoimmune diseases: for examples, lupus erythematosus, antiphospholipid syndrome (anti-nuclear, anti-DNA, antiphospholipid antibodies), celiac disease (gliadin and transglutaminase antibodies), primary Sjögren, anti-SSB [anti-SSA (Sjögren syndrome antigen A and B)], thyroid antibodies in Hashimoto's encephalopathy (Thyroid peroxidase, thyroglobulin antibodies) (50, 51). Paraneoplastic causes were often associated with weight loss, male gender, and older age (52) and were more associated with anti-CRMP5/CV2 (collapsin response-mediator protein-5) and anti-Hu/ANNA-1. Although it did not emerge from our results, it is important to note that paraneoplastic chorea can be associated with peripheral neuropathy and orolingual facial dyskinesia, the latter being typically described in anti-NMDA (N-methyl-D-aspartate) receptor encephalitis. We recommend considering the investigation of autoimmune disorders, paraneoplastic, or not, at the latest in second-line because of its potential therapeutic implications. During the review process, it emerges that even if they do not appear first-line statistically, it would make good sense in some circumstances (e.g., unexplained weight loss) include them as in the first-line testing.

Although generally manifesting in pediatric age, Niemann Pick C disease can be observed in adults given the possibility of mild late-onset forms and the increasing survival of pediatric patients by reason of the improvement of the available treatments. Movement disorders may be the first sign in this disease (53), in which cerebellar ataxia, dystonia, vertical supranuclear gaze palsy dominate the picture, with chorea being less common. These signs are associated with psychiatric syndromes (depression, psychosis) and constant cognitive impairment in adults. Splenomegaly may not be present in adult-onset form. Due to the available treatment, this disease should not be misdiagnosed: one should thus consider ordering plasma metabolites (cholestane-3β, 5α, 6β-triol, lyso-sphingomyelin isoforms, and bile acid metabolites), supplementing molecular genetic studies (54).

#### Imaging

MRI should include iron-sensitive sequences such as echo sequences, susceptibility-weighted imaging, or T2^*^-weighted sequences. The presence of iron accumulation in the basal ganglia leads to the diagnosis of a large group of clinically and genetically heterogeneous diseases included in the Neurodegeneration with brain Iron Accumulation (NBIA) group (55). This group includes four disorders that might manifest as HD phenocopy.

Neuroferritinopathy (NF) is characterized by adult-onset chorea with dystonia or parkinsonism, while cerebellar, psychiatric, and cognitive signs are less prominent. MRI also reports cavitary lesions in NF.

Aceruloplasminemia (ACP) may also present with an HD-like phenotype with ataxia and signs of systemic disorders such as diabetes and retinal degeneration. Although our algorithm suggest considering ACP and Wilson's disease in second-line testing, it would not be unreasonable to run them at first line, given that they are treatable conditions. Serum ceruloplasmin and copper levels are low, but in contrast to Wilson's disease, urine copper levels are normal.

In their atypical forms, pantothenate kinase-associated neurodegeneration (PKAN) and phospholipase A2-associated neurodegeneration (PLAN, also called Neuroaxonal dystrophy NAD), might manifest with adult-onset chorea associated with parkinsonism, dystonia, and cognitive impairment.

### Additional Suggestions

To ensure exhaustive and comprehensive coverage, five paraclinical tests were added following the results of the surveys and voted on afterwards by the participant experts, including the 23 experts from the disease group “Chorea” of ERN-RND. Muscle biopsy, toxoplasmosis and Lyme disease analyses were added to the 3rd-line tests (respectively, 88, 48, 52% of votes). By a narrow margin, B12 metabolism (including holotranscobalamin, methylmalonic acid, and homocysteine) was rated as a 1st-line test (48% 1st-line, 44% 2nd-line). The parathyroid test was considered as a 2nd line test (48%).

The committee suggested these two gene mutations that were not incorporated beforehand should also be considered (56). A Saudi Arabian family with juvenile onset chorea, dementia, and seizures should suggest the *FRRS1L* mutation. Chorea, dementia, and the association of hypodontia, ataxia, hypogonadotropic hypogonadism, and white matter lesions may suggest a *RNF216* mutation.

We intended to validate red flags and a workout that would be feasible in the majority of HD phenocopies in DCP. Surveys have been shown to be a useful tool for investigating clinical practices (57, 58). However, some limitations should be mentioned. The participation rate was around 25%, consequently a margin of error of around 20% was considered to reach a confidence level of 95%. Most of the physicians were European (90% for the red-flag survey, and 84% for the paraclinical tests survey). Therefore, the results would first and foremost reflect European practices. Additionally, although we sent general and individual reminder emails, eight physicians signed up but did not complete both surveys.

Discrepancies between literature and physicians could have several explanations. Physicians were limited to suggesting up to three diagnoses. An example of this case could be the red flag for “severe dystonia” or “seizures” where the votes were spread among many diagnoses. Rarer red flags may also be recognized by fewer physicians. The long period of time of the review could include different availability of tests or clinical practices around the world. Thus, some tests, such as anti-streptolysin, HIV, and syphilis tests, could be considered 1st-line according to local epidemiology.

## Limitations and Conclusion

Here, we provide clinical expertise-based diagnosis guidelines for HD phenocopy patients, allowing for a rational use of resources in the diagnostic process.

However, during the review process, some issues emerged that had not appeared in either the literature review nor in the surveys. This is the case, for example, of whole exome sequencing (WES), which is still an infrequently employed, but growing practice. Recommendations for ordering WES are work in progress. Similarly, for SCAs, our questionnaires did not aim to classify them specifically, even if the literature shows that their frequency is unevenly distributed. It also appears that the treatability of the condition would suggest considering some paraclinical examinations as first-line, because of the consequences of delaying a potential proven and efficacious treatment (e.g., lowering copper load), it was thus highlighted in the discussion. Finally, some topical articles did not appear in the literature review because our selection of articles was based first on automatic review of keywords and then titles before experts analyzed the selected abstract. The automatic selection might have restricted the selection of abstract. In addition, some relevant reviews, like (59), appeared a month after the literature review was completed and were therefore not mentioned despite their obvious interest. Nevertheless, our article listed more red flags that any previous reviews and thus presumably did not miss many useful red flags. We do not claim to be exhaustive, even if we aim to reach a compromise between completeness and pragmatism reflecting daily clinical practice of clinicians in the 2020s. Such an approach should be useful in the future for other rare diagnoses as well. It is likely that certain rare disease entities are currently unidentified; this may well change in the course of the next coming years thanks to the progress in technologies to identify diseases.

## Contributors

Australia: Mark Walterfang (Melbourne Neuropsychiatry Centre).

China: Zhi-Ying Wu (Department of Neurology, School of Medicine Zhejiang University).

Czech Republic: Jiri Klempir (Department of Neurology, Charles University, Prague), Jan Roth (Department of Neurology, Charles University, Prague).

Denmark: Anette Torvin Møller (University Hospital of Aarhus, Aarhus).

France: Alexandra Durr (Centre de Recherche de l'Institut du Cerveau et de la Moelle Epinière, Paris), Perrinne Charles (Department of Neurology, hôpital Pitié-Salpêtrière, Paris), Adriana Prundean (Department of Neurology, Centre Hospitalier Universitaire, Angers), Clarisse Scherer (Department of Neurology, Centre Hospitalier Universitaire, Angers), Christine Tranchant (Department of Neurology, hôpitaux universitaires de Strasbourg).

Germany: Ludger Schöls (Department of Neurodegeneration, University of Tübingen).

Italy: Marina Frontali (Institute of Neurobiology and Molecular Medicine, CNR, Rome), Lorenzo Nanetti (Unit of Genetics of Neurodegenerative and Metabolic Diseases, Istituto Neurologico Carlo Besta, Milan).

Netherlands: Mayke Oosterloo (Department of Neurology, Maastricht University Medical Center), Corien Verschuuren (Department of Genetics, University Medical Center Groningen), Bart van de Warrenburg (Department of Neurology, Radboud University Medical Center, Nijmegen).

Poland: Dec-Cwiek Malgorzata (Department of Neurology, Medical College, Kraków).

Portugal: Carolina Garrett (Departamento de Neurociências Clínicas e Saúde Mental, Porto), João Massano (Departamento de Neurociências Clínicas e Saúde Mental, Porto).

Romania: Tibre Vasile (Department of Neurology, UMF Cluj-Napoca).

Spain: Esteban Muñoz (Hospital Clínic de Barcelona).

United Kingdom: Roger Barker (Department of Clinical Neurosciences, University of Cambridge).

USA: Susan Perlman (Department of Neurology, David Geffen School of Medicine, Los Angeles).

## Data Availability Statement

The raw data supporting the conclusions of this article will be made available by the authors, without undue reservation.

## Author Contributions

A-CB-L and GL supervised the elaboration of this study and guidelines. QN supervised the online surveys and analyzed the results. QN, A-CB-L, and KY selected the studies to be analyzed. QN and A-CB-L wrote the original draft preparation, review, and editing the manuscript. JO, J-MB, CM, CS, and LH reviewed and edited the manuscript. All authors contributed to the article and approved the submitted version.

## Funding

This work was supported by ANR-17-EURE-0017 and the national centre of reference for Huntington's disease (French Ministry of Health).

## Conflict of Interest

The authors declare that the research was conducted in the absence of any commercial or financial relationships that could be construed as a potential conflict of interest.

## Publisher's Note

All claims expressed in this article are solely those of the authors and do not necessarily represent those of their affiliated organizations, or those of the publisher, the editors and the reviewers. Any product that may be evaluated in this article, or claim that may be made by its manufacturer, is not guaranteed or endorsed by the publisher.
