# Corrigendum: Combining Literature Review With a Ground Truth Approach for Diagnosing Huntington's Disease Phenocopy

**DOI:** 10.3389/fneur.2022.891800

**Published:** 2022-03-30

**Authors:** Quang Tuan Rémy Nguyen, Juan Dario Ortigoza Escobar, Jean-Marc Burgunder, Caterina Mariotti, Carsten Saft, Lena Elisabeth Hjermind, Katia Youssov, G. Bernhard Landwehrmeyer, Anne-Catherine Bachoud-Lévi

**Affiliations:** ^1^AP-HP, Hôpital Henri Mondor-Albert Chenevier, Centre National de Référence Maladie de Huntington, Service de Neurologie, Créteil, France; ^2^Univ Paris Est Creteil, INSERM U955, Institut Mondor de Recherche Biomédicale, Laboratoire de Neuropsychologie Interventionnelle, Creteil, France; ^3^Département d'Etudes Cognitives, École normale supérieure, PSL University, Paris, France; ^4^Movement Disorders Unit, Institut de Recerca Sant Joan de Déu, CIBERER-ISCIII, Barcelona, Spain; ^5^European Reference Network for Rare Neurological Diseases (ERN-RND), Tübingen, Germany; ^6^Siloah and Department of Neurology, Department of Clinical Research, Swiss Huntington's Disease Centre, University of Bern, Bern, Switzerland; ^7^Unit of Genetics of Neurodegenerative and Metabolic Diseases, Carlo Besta Neurological Institute IRCCS Foundation, Milan, Italy; ^8^Department of Neurology, Huntington Center North Rhine-Westphalia, Ruhr-University, St. Josef-Hospital, Bochum, Germany; ^9^Department of Neurology, Rigshospitalet, Danish Dementia Research Centre, Clinic of Neurogenetics, Copenhagen University Hospital, Copenhagen, Denmark; ^10^Department of Neurology, University of Ulm, Ulm, Germany

**Keywords:** Huntington's disease, chorea, phenocopy, diagnosis, differential diagnosis, guidelines, daily clinical practice

In the original article, there was a mistake in Figure 2 and Figure 3 as published. The figures were swapped incorrectly.

**Figure 2:**
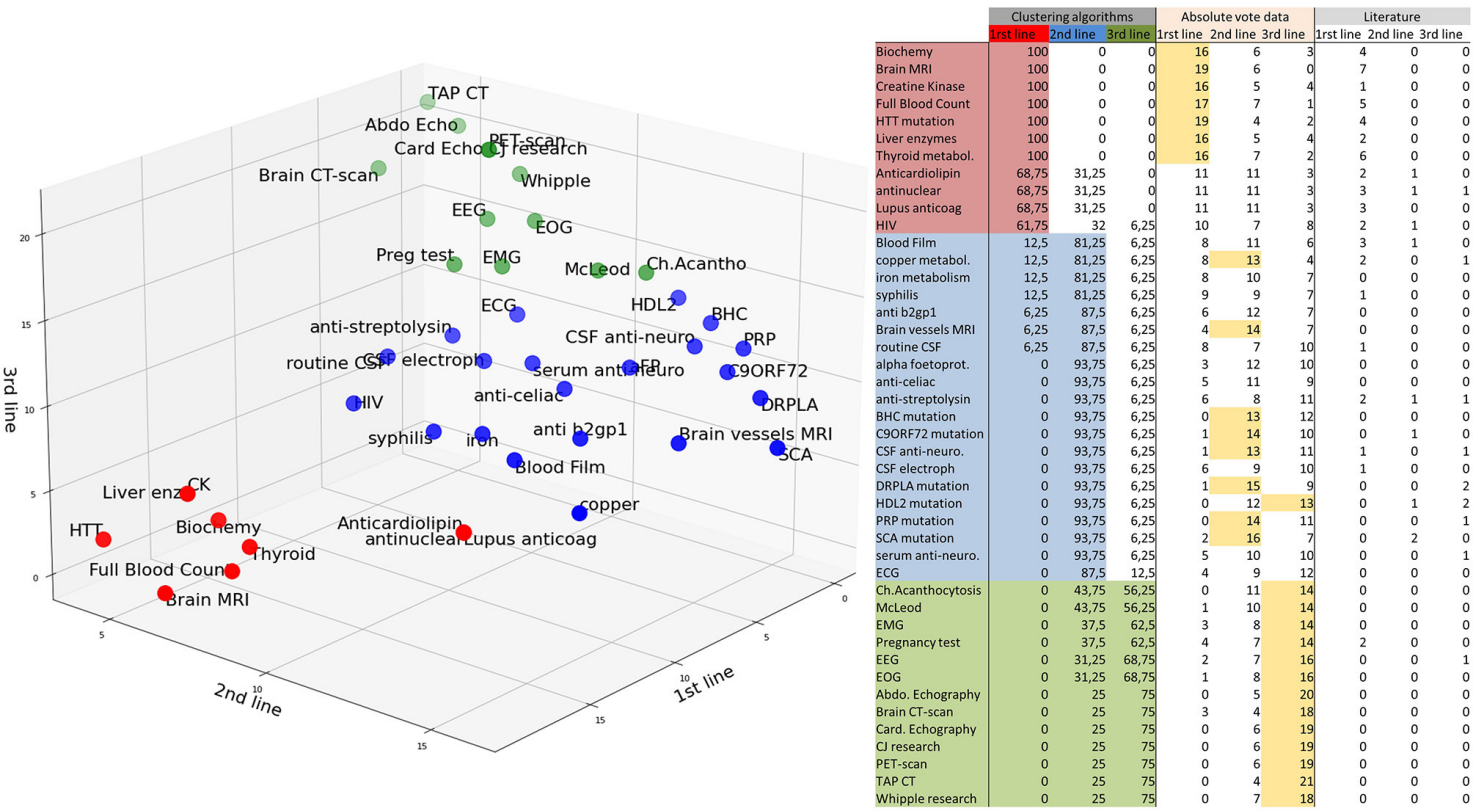
Classification of the paraclinical tests. 1st-line: red, 2nd-line: blue, 3rd-line: green. The results of the clustering algorithms are summed in the left columns (results in % of runs of the algorithms). Absolute numbers of votes are reported in the middle columns as “Absolute vote data.” The majority vote (>50% of 25 votes) is highlighted in yellow. Literature results are summarized in the right columns. McLeod: exclusion of the McLeod Kell phenotype by a blood bank (weak Kell antigens and, if available, no reaction with anti-Kx). Ch.acanthocytosis: Western blot for chorein protein.

**Figure 3:**
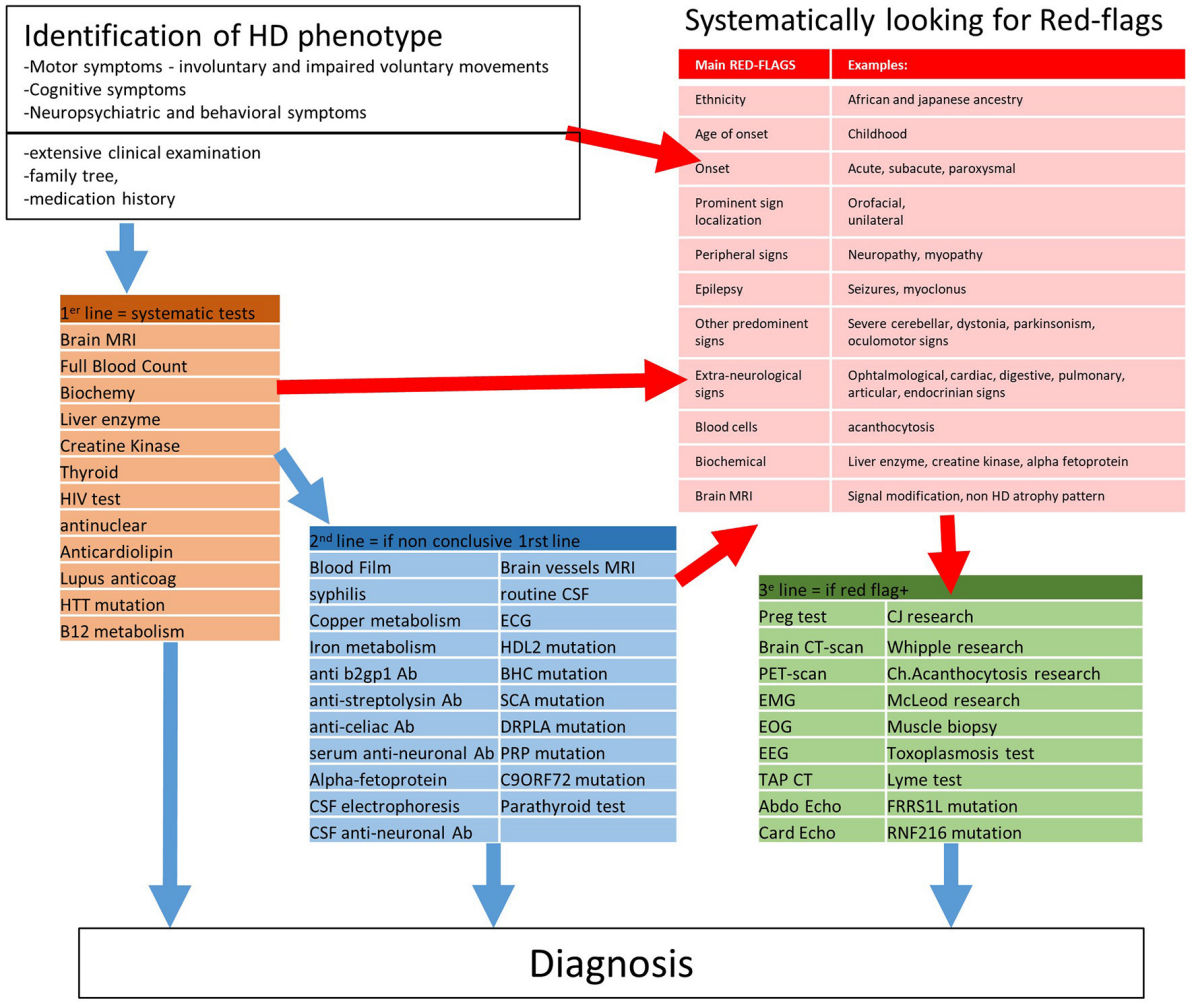
Proposition of a strategy to diagnose HD phenocopy. Ab, Antibodies.

The authors apologize for this error and state that this does not change the scientific conclusions of the article in any way. The original article has been updated.

## Publisher's Note

All claims expressed in this article are solely those of the authors and do not necessarily represent those of their affiliated organizations, or those of the publisher, the editors and the reviewers. Any product that may be evaluated in this article, or claim that may be made by its manufacturer, is not guaranteed or endorsed by the publisher.

