# Supplementary material for: Combining Literature Review With a Ground Truth Approach for Diagnosing Huntington's Disease Phenocopy
Source: Front Neurol. 2022 Feb 10;13:817753. doi: 10.3389/fneur.2022.817753 (PMC8866848; doi:10.3389/fneur.2022.817753)
Supplement: Supplementary file 1 [file Table_1.DOCX]

**Identification of studies via Pubmed**

Records removed *before screening*:

Non-English articles (n = 279)

Records identified from:

Databases (n=4361):

4361

keywords: "Chorea" or "Huntington" and “Diagnosis

**Identification**

Records marked as ineligible by automation tools (n = 4031)

Records screened

(n = 4082)

Reports not retrieved

(n = 2 ):

1 comment to editor, 1 not retrievable

Reports sought for retrieval

(n = 51)

**Screening**

Reports excluded based on the abstracts review:

Specifically about Huntington disease (n=29, update, 5 mutation, 11 diagnosis, 8 review, 2 pathology, 1 philosophy, 1 nursing)

Reports assessed for eligibility by two raters independently

(n = 49)

Studies included in review

(n = 20)

**Included**

Supplemental materials: PRISMA 2020 flow diagram from <http://www.prisma-statement.org/>
